# Physical Education on the Beach: An Alternative Way to Improve Primary School Children’s Skill- and Health-Related Outcomes during the COVID-19 Pandemic

**DOI:** 10.3390/ijerph19063680

**Published:** 2022-03-19

**Authors:** Maria Chiara Gallotta, Giovanna Zimatore, Ludovica Cardinali, Lavinia Falcioni, Valerio Bonavolontà, Davide Curzi, Laura Guidetti, Carlo Baldari

**Affiliations:** 1 Department of Physiology and Pharmacology “Vittorio Erspamer”, Sapienza University of Rome, 00185 Rome, Italy; 2 Department of Theoretical and Applied Sciences, eCampus University, 22060 Novedrate, Italy; giovanna.zimatore@uniecampus.it (G.Z.); carlo.baldari@uniecampus.it (C.B.); 3 Department of Movement, Human and Health Sciences, University of Rome “Foro Italico”, 00135 Rome, Italy; ludovica.cardinali@uniroma4.it (L.C.); laviniafalcioni@gmail.com (L.F.); 4 Department of Basic Medical Sciences, Neuroscience and Sense Organs, School of Medicine, University of Bari “Aldo Moro”, 70124 Bari, Italy; valerio.bonavolonta@uniba.it; 5 University Niccolò Cusano, 00166 Rome, Italy; davide.curzi@unicusano.it (D.C.); laura.guidetti@unicusano.it (L.G.)

**Keywords:** COVID-19 pandemic, physical education, beach, school context, motor performance, health, fitness, physical activity level

## Abstract

The COVID-19 restrictions could preclude children from participating in physical education (PE) interventions. This study aimed to evaluate the efficacy of a PE intervention conducted on the beach on children’s skill- and health-related outcomes, as a possible alternative PE intervention that could be also applied during the COVID-19 pandemic. The study involved 106 primary school children, randomly assigned to the traditional indoor (TI) intervention or to the experimental outdoor (EO) intervention. The intervention period lasted 4 months and consisted of two 1-h sessions per week. Intervention was conducted just before the beginning of the COVID-19 pandemic. Children’s anthropometric parameters (height, weight, BMI, body fat percentage, and abdominal circumference), fitness parameter (VO_2peak_), health parameters (resting heart rate, and systolic and diastolic blood pressure), gross motor coordination, and physical activity level were assessed before and after intervention. Both groups significantly improved fitness and motor coordination but worsened some anthropometric parameters (weight, abdominal circumference) after the intervention period. The EO group showed a higher increase of gross motor coordination than the TI group. Results of this study demonstrated that children benefited from a well-structured PE intervention conducted in the natural environment of the beach improving physical fitness and gross motor coordination. Therefore, planning outdoor PE interventions could be an alternative and safe way to encourage and implement physical activity at school during the particular period of COVID-19 pandemic.

## 1. Introduction

The increase of physical inactivity over the past decades is one of the main causes in the development of obesity and its complications from a very young age [1]. The lack of adequate level of physical activity in children is associated with increasing prevalence of cardiovascular risk factors, such as obesity, hypertension, dyslipidaemia, insulin resistance, and vascular inflammation, which can be modified by changes in lifestyle behaviors such as physical activity and diet [2]. Moreover, studies reported that children’s increase of sedentary behaviors and the decrease of physical activity caused a substantial decline in their motor competence [3,4,5,6]. It was well demonstrated that regular participation in physical activity induced positive effects on children’s physical and mental health improving health-related quality of life, physical and social functioning [7]. The World Health Organization (WHO) recommends that young people should do at least an average of 60 min of moderate-to-vigorous physical activity (MVPA) daily, including aerobic exercises and activities to increase and maintain muscular strength, flexibility, and bone health [8,9]. However, a large proportion of young people engage in less physical activity necessary for good health [8]. Recent studies conducted on Italian populations report that more than 18% of Italian children are sedentary [6,10,11,12]. In this context, physical education (PE) appears to be an ideal instrument to promote physical activity participation, since many children and adolescents can be reached, to encourage young people to establish a long-lasting healthy lifestyle and to improve their motor performance [13,14]. Schools could provide opportunities to children and adolescents to be physically active during PE classes, providing them an opportunity to train in an appropriate manner and to participate in adequate PE interventions [13,14,15]. PE interventions are usually conducted in indoor spaces (school gyms). Nevertheless, the COVID-19 restrictions such as the closure of schools and the cancellation of youth sports and PE classes may prevent children from participating in PE interventions [16]. Considering that COVID-19 is spread by direct contact via droplets, the airborne transmission of SARS-CoV-2, should be considered dangerous, especially in indoor environments [17]. The literature suggests that indoor environments, such as gyms, have the greater risk of infection than outdoor environments due to the possible build-up of airborne virus-carrying droplets, inefficient ventilation, and the higher stability of the virus in indoor air [18,19]. Thus, different strategies were applied in Italy in order to prevent or limit contagions during COVID-19 PE practices. It was mandatory to pay attention to dimensions of curricular spaces to allow interpersonal distance of at least 2 meters while doing activities. Individual physical activities were privileged while team sport activities were limited or forbidden. The use of PE tools and sports equipment was limited to personal use only and they should disinfected at the end of each PE lesson. Moreover, the need to wear masks during physical activities, led necessarily to PE lessons with light exercises [20]. Therefore, the necessity of exercising outdoors has become evident following the COVID-19 outbreak. Open spaces could remedy the problem of insufficient spaces for PE, that frequently caused reduction or suspension of PE practical lessons. Outdoor physical exercises no longer needed to use masks; therefore, children could exert respecting the MVPA intensity range [8,9]. Moreover, outdoor sports and physical activities allowed children to benefit from the physical exercise opportunities offered by the natural environment, promoting physical and psychological health and well-being [21,22], positively affecting physical activity level [23], motor skills development [24], fitness level increase [25], and cognitive and social development [22]. Outdoor PE activities could also favor the body image perception and representation [26]. Thus, planning outdoor PE interventions could be an alternative and safe way to encourage and implement physical activity at school, favoring the achievement of recommended levels of physical activity to improve school children’s skill- and health-related outcomes.

The Italian peninsula, located in the middle of the Mediterranean Sea, comprises a continental northern sector, a peninsular central-southern sector, two large islands (Sicily and Sardinia) and various archipelagos and minor islands for a total of 20 regions of which 15 are coastal regions [27]. As a peninsula, it is characterized by a large number of coastal areas and beaches that represent an ideal outdoor environment for practicing physical activity.

Thus, this study was aimed to propose an alternative way to the traditional PE intervention. The aim of this study was to verify the feasibility and to evaluate the efficacy of a PE intervention conducted in a natural environment (the beach) on children’s skill- and health-related outcomes, as a possible alternative and safe PE intervention that could be applied also during the COVID-19 pandemic.

## 2. Materials and Methods

### 2.1. Participants

One hundred and six primary school children aged 7–11 years volunteered to participate in this study. The sample included subjects from two different schools in Anzio, a small town near the beach in the south of Rome (Italy). Sixty-four participants were randomly assigned to an experimental outdoor PE program (EO) lasting 4 months. The remaining 42 participants were assigned to a traditional indoor PE program (TI) of the same duration and frequency (Table 1).

The Sapienza University Ethical Committee approved this investigation (Rif 5500 Prot. 1070/19) in accordance with the ethical standards laid down in the 1964 Declaration of Helsinki and its later amendments. Written informed consent forms were obtained from both parents and children prior to study participation.

### 2.2. Assessment Methods

#### 2.2.1. Anthropometric Parameters Assessment

Children’s height, weight, body mass index (BMI), abdominal circumference, and body fat were assessed before and after intervention. The measurements were taken according to the standard procedures described by Lohman et al. [28]. Weight and height were measured using a scale and a stadiometer to the nearest 0.5 kg and 0.1 cm, respectively. Children’s BMI was calculated as weight in kg divided by the square of height in meters. Abdominal circumference was measured at the level of the greatest anterior extension of the abdomen in a horizontal plane, when the subject stood. The measurement was made using a tape to the nearest 0.1 cm. Body fat percentage was measured by foot-to-foot bioelectrical impedance analyzers (Body Fat Monitor Scale BF-625, Tanita Corporation of America Inc., Arlington Heights, IL, USA; Téfal Bodymaster Vision, Téfal, Rumilly, France).

#### 2.2.2. Health Parameters Assessment

Resting heart rate and resting blood pressure were measured before and after intervention. Measurements were conducted after children laid on mats for 10–15 min listening to relaxing music. The heart rate was assessed using a heart rate monitor (S610i; Polar Electro Oy, Kempele, Finland). The lowest value stabilized by the heart rate monitor was considered for resting heart rate. The evaluation of the blood pressure was performed in the left arm. The measurements were performed, using a sphygmomanometer, stethoscope, and cuff suitable for the children’s brachial perimeter.

#### 2.2.3. Motor Performance Assessment

Children’s aerobic fitness and gross motor coordination were assessed before and after intervention.

The Pacer test was used to assess aerobic power and cardiovascular endurance [29]. Participants were instructed to run back and forth between two lines spaced 20-m apart, at increasing speed. The test was continued until the participants reached exhaustion or could not complete the laps twice continuously within the required time limit. The Pacer test was reported as a valid measurement of aerobic fitness (R^2^ = 0.80) [29,30]. The equation of Matsuzaka et al. [30] was used to predict VO_2peak_ values (mL·kg^−1^·min^−1^) from the PACER scores (laps). The equation had high evidence of validity [R^2^ = 0.81] [30].

All four subtests of the Körperkoordinations Test für Kinder (Body Coordination Test for Children, referred to as KTK) were used to evaluate children’s motor coordination [31]. All the tests assess gross motor skills, body control, and coordination, mainly dynamic balance skills [32,33]. These tests were:

The balance beam test assesses the stability of balance in the forward and backward paths. Participants were instructed to walk backward for three times along each of three balance beams of progressively decreasing width. The score was the number of correctly performed steps (maximum eight steps per trail) for each balance beam.

The jumping laterally test assesses the speed of execution with alternating jumps. Participants were instructed to jump laterally as many times as possible over a slat in 15 s. The score was the number of correctly performed jumps.

The hopping on one leg over an obstacle test assesses the coordination of the lower limbs and the dynamic power/force. Participants were instructed to hop on one leg over an increasing pile of pillows after a short run-up. Three, two, or one point(s) were/was awarded for successful performance on the first, second, and third trial, respectively. A maximum of 39 points could be scored for each leg. The sum score was computed.

The shifting platforms test assesses laterality and space–time structure. Participants were instructed to move across the floor in 20 s by stepping from one plate to the next. The score was the number of correctly performed relocations.

The test–retest reliability coefficient for the raw score on the total test battery was previously reported as 0.97, while corresponding coefficients for individual tests ranged from 0.80 to 0.96. Both factor analysis and inter-correlations indicated acceptable construct validity [31].

A standardized warm-up of 10 min running, jumping and stretching exercises preceded the test assessment. Before each subtest, children received an oral explanation and a demonstration about test procedure.

#### 2.2.4. Physical Activity Level Assessment

Children’s physical activity level was assessed before and after intervention by the Italian version of the Physical Activity Questionnaire for Older Children (PAQ-C-It) [34]. It is a 7-day recall, self-administered instrument. It is composed by nine questions about games and sports, physical activities at school, and those during leisure time, including the weekend. Each question is scored from 1 to 5, with the final score obtained through the means of the question scores. It was previously reported that the PAQ-C-It had acceptable to good reliability (alpha 0.70 to 0.83) and it had significant concurrent validity with the objectively measured MVPA (rho = 0.30, *p* < 0.05) [34].

### 2.3. Intervention Programmes

The intervention period lasted 4 months. Intervention was conducted just before the beginning of the COVID-19 pandemic. Experimental interventions consisted of two 1-h sessions per week, for a total of 30 PE lessons for each intervention group. PE interventions differed in environment/context where they were conducted, as well as in type and mode of physical activities in which children participated but they were equivalent in terms of structure, total duration and individual perceived exertion. The individual perceived exertion of both intervention programs was monitored using the OMNI scale [35] to avoid possible differences between the two interventions. Both interventions were designed and conducted by the same specialist PE teacher. Each lesson of both interventions included 15 min of warm-up, 35 min of MVPA within a range of 5 < RPE < 8 [36] and 10 min of cool-down and stretching. During each MVPA session, children reported their OMNI RPE measures. They verbally indicated the corresponding number after looking at the scale in order to have an indication of how hard the exertion felt during the exercise session.

The TI was conducted in the school gym. It was planned to promote children’s fitness and health, sensory-motor, communicative, and social development. It was designed to improve primarily flexibility, strength, and endurance by circuit training for cardiovascular health [37].

The EO was conducted on the beach with the use of various conventional and unconventional tools (e.g., beams derived from beach cabins bases to perform balance exercises). It consisted in a lot of aerobic activities such as walking, running, jumping, walking on their hands and knees, rolling on the beach, creeping, and climbing over. Moreover, different kinds of walking, like walking forward, backward, sideways, tip toeing, striding, on their heels, walking varying the position of the arms, with closed eyes, walking fast and slowly, and so on, were proposed.

All these exercises were conducted in multiple different, playful ways, one by one or in a team.

### 2.4. Data Analysis

All results were expressed as mean ± standard deviation. An unpaired *t*-test comparison was firstly conducted on pre-test values of each variable to check for any difference between the two groups before the intervention. A mixed-model analysis of variance (ANOVA) was performed for each measured parameter with group and gender as between-participants factors, time as within-participants factor. The main effects of group (two levels, EO vs. TI), gender (two levels, girls vs. boys), and time (two levels, pre vs. post) were assessed, as well as the interaction between them (time × group, gender × group, time × gender). Where significant main effects were observed, Bonferroni post hoc analysis was used to aid interpretation of these interactions. Effect size was also calculated using Cohen’s definition of small, medium, and large effect size (as partial η^2^ = 0.01, 0.06, 0.14, respectively) [38]. Finally, subgroup analyses were performed by means of planned pairwise comparisons (*t*-tests) to examine the intervention effect on each variable by participants’ gender and group. Statistical significance was defined as *p* ≤ 0.05.

## 3. Results

Differences in the baseline variables of the EO group and the TI group were verified (*p* < 0.05), but no significant differences between the two groups were revealed.

The main effect of time revealed that children’s weight (F_1,102_ = 135.75, *p* < 0.0001, η^2^ = 0.571) (32.9 ± 10.5 kg vs. 34.8 ± 11.3 kg) and abdominal circumference (F_1,100_ = 16.74, *p* < 0.0001, η^2^ = 0.143) (62.8 ± 10.7 cm vs. 64.0 ± 10.9 cm) significantly increased after intervention. The main effect of gender revealed that girls had higher %FM than boys (F_1,101_ = 8.26, *p* = 0.005, η^2^ = 0.076) (26.3 ± 5.0 vs. 22.7 ± 7.5, respectively). The time × group interaction (F_1,102_ = 10.86, *p* = 0.001, η^2^ = 0.096) revealed that only the EO group significantly increased BMI after intervention (Table 2).

**Figure 1:**
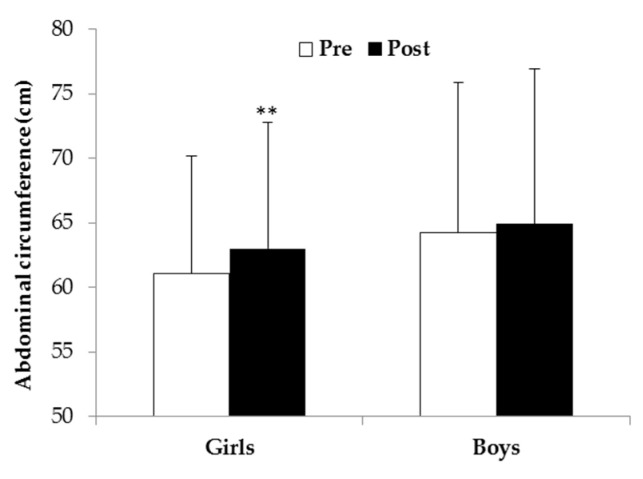
Abdominal circumference of girls and boys before (Pre) and after (Post) intervention (** *p* < 0.001).

The time × gender interaction (F_1,100_ = 5.15, *p* = 0.025, η^2^ = 0.049) revealed that girls significantly increased their abdominal circumference after intervention (Figure 1).

The main effect of gender (F_1,91_ = 4.05, *p* = 0.047, η^2^ = 0.043) revealed that girls had higher resting HR than boys (82.5 ± 12 bpm vs. 79.4 ± 10.4 bpm, respectively). The time × group interaction (F_1,91_ = 17.64, *p* < 0.0001, η^2^ = 0.162) revealed that the EO group significantly increased resting HR, while the TI group significantly decreased resting HR rate after intervention (Table 2).

**Figure 2:**
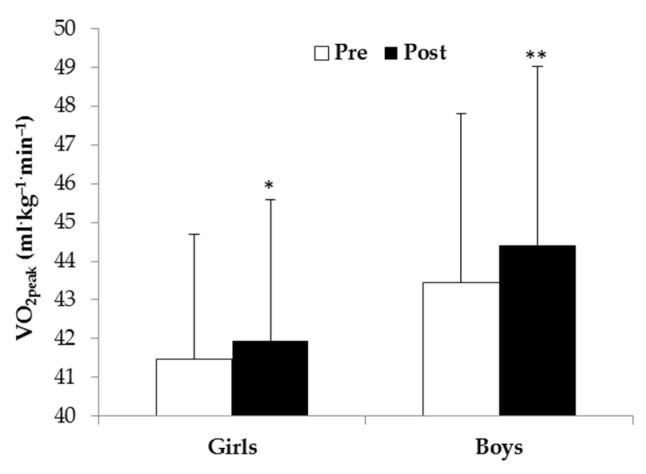
VO_2peak_ of girls and boys before (Pre) and after (Post) intervention (* *p* = 0.01, ** *p* < 0.001).

The main effect of time revealed that children’s VO_2peak_ (F_1,102_ = 27.70, *p* < 0.0001, η^2^ = 0.214) (42.3 ± 4.0 mL·kg^−1^·min^−1^ vs. 43.1 ± 4.4 mL·kg^−1^·min^−1^) and laps number (F_1,101_ = 82.91, *p* < 0.0001, η^2^ = 0.451) (9.5 ± 4.7 num vs. 14.5 ± 8.1 num) significantly increased after intervention. The main effect of group (F_1,101_ = 4.48, *p* = 0.037, η^2^ = 0.042) revealed that the EO group performed a greater number of laps than the TI group (13.0 ± 8.2 num vs. 10.5 ± 4.6 num, respectively). The main effect of gender revealed that girls had lower VO_2peak_ (F_1,102_ = 6.56, *p* = 0.012, η^2^ = 0.060) (41.7 ± 3.4 mL·kg^−1^·min^−1^ vs. 43.7 ± 4.6 mL·kg^−1^·min^−1^, respectively) and performed a lower number of laps (F_1,101_ = 4.75, *p* = 0.032, η^2^ = 0.045) (10.8 ± 5.4 num vs. 13.2 ± 8.3 num, respectively) than boys. The time × group interaction (F_1,101_ = 8.57, *p* = 0.004, η^2^ = 0.078) revealed that the EO group showed a higher increase of laps number than the TI group after intervention (Table 2). The time × gender interaction (F_1,102_ = 5.05, *p* = 0.027, η^2^ = 0.047) revealed that boys had a higher increase of VO_2peak_ than girls after intervention (Figure 2).

**Figure 3:**
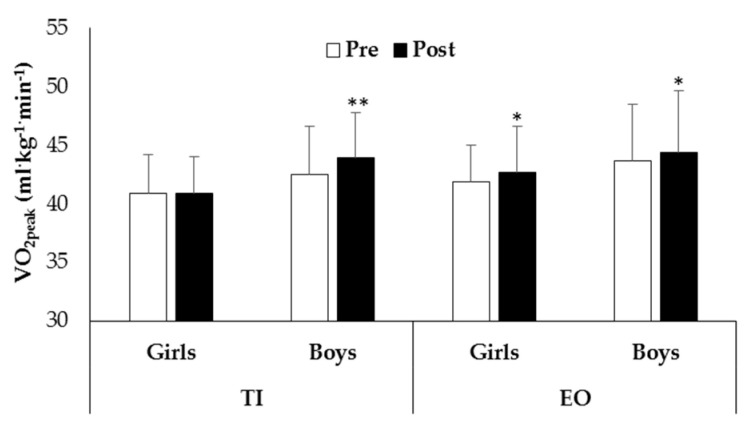
VO_2peak_ of girls and boys before (Pre) and after (Post) intervention in experimental outdoor intervention (EO) and traditional indoor intervention (TI) (* *p* = 0.01, ** *p* = 0.001).

Moreover, subgroup analyses revealed that all boys and only girls of the EO group significantly increased VO_2peak_ after intervention (Figure 3).

**Figure 4:**
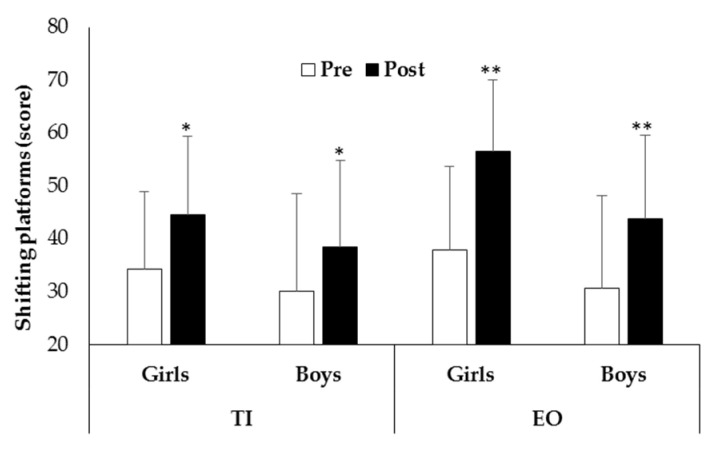
Shifting platforms test of girls and boys before (Pre) and after (Post) intervention in experimental outdoor intervention (EO) and traditional indoor intervention (TI) (* *p* < 0.01, ** *p* < 0.001).

The main effect of time revealed that children’s shifting platforms test (F_1,101_ = 78.03, *p* < 0.0001, η^2^ = 0.436) (30.6 ± 9.2 score vs. 38.3 ± 10.0 score), balance beam test (F_1,101_ = 123.60, *p* < 0.0001, η^2^ = 0.550) (33.2 ± 16.4 score vs. 46.8 ± 16.3 score), jumping laterally test (F_1,100_ = 119.24, *p* < 0.0001, η^2^ = 0.544) (22.6 ± 15.2 score vs. 41.6 ± 20.7 score), and hopping on one leg test (F_1,101_ = 123.61, *p* < 0.0001, η^2^ = 0.550) (36.5 ± 18.2 score vs. 48.7 ± 16.2 score) significantly increased after intervention. The main effect of group (F_1,101_ = 3.10, *p* = 0.049, η^2^ = 0.038) revealed that the EO group had a higher balance beam test score than the TI group (42.2 ± 18.2 score vs. 37.0 ± 16.7 score, respectively). The main effect of gender (F_1,101_ = 7.55, *p* = 0.007, η^2^ = 0.070) revealed that girls had a higher balance beam test score than boys (44.0 ± 17.0 score vs. 36.1 ± 17.7 score, respectively). The time × group interaction revealed that the EO group showed a greater increase of balance beam (F_1,101_ = 5.61, *p* = 0.020, η^2^ = 0.053) and jumping laterally (F_1,101_ = 6.79, *p* = 0.011, η^2^ = 0.064) tests than the TI group after intervention (Table 2). Moreover, subgroup analyses revealed that the shifting platforms test of all boys and girls significantly increased after intervention, however girls of the EO group showed the greater increase (Figure 4).

Finally, the main effect of group (F_1,101_ = 8.84, *p* = 0.004, η^2^ = 0.080) revealed that the EO group had a greater PAQ-C-It score than the TI group (2.6 ± 0.6 score vs. 2.3 ± 0.7 score, respectively).

## 4. Discussion

The COVID-19 pandemic induced changes in PE in order to prevent or reduce contamination in teachers and students. Sports and physical activities increase droplet speed and output; thus, students have to use masks during physical activities, gyms should be constantly ventilated, physical contact should be limited, and sports equipment should be disinfected at the end of each PE lesson [39,40]. Therefore, open spaces seem to be better than confined spaces and the safest option where to conduct the PE interventions [39]. Thus, this study was aimed to propose and verify the efficacy of an alternative and safe way to the traditional PE intervention that could be also applied during the COVID-19 pandemic. We investigated the efficacy of a PE intervention conducted in a natural environment (the beach) on schoolchildren’s anthropometric and health-related parameters, physical fitness, gross motor coordination and physical activity level.

The lack of a favorable impact of both interventions on children’s anthropometric parameters confirmed that PE interventions could induce limited positive effects on body weight, BMI and FM% [13]. An average of 60 min of MVPA daily is necessary to reduce adiposity in children and adolescents [8]. Although we respected in both interventions these guidelines in terms of intensity and duration of physical activity, we could not fully comply with them in terms of weekly frequency for reasons linked to the scheduling of school activities. This aspect could have limited the achievement of beneficial effects on children’s body composition.

TI group significantly decreased resting HR after intervention, confirming that increased structured physical activity is related to lower resting HR [41,42]. On the contrary, the EO group significantly increased resting HR after intervention although previous studies suggested that natural environments may give rise to stress-buffering influences [43]. It was possible that a new situational condition (physical activity on the beach with a specialist PE teacher) could negatively influence resting HR. Girls of our study had higher resting HR and lower VO_2peak_ than boys confirming that resting HR is inversely associated with aerobic fitness [44].

Moreover, findings of the present study suggested that a well-structured physical activity program, conducted by a specialist PE teacher, led to an increase in children’s aerobic fitness. In fact, both boys and girls of the EO group and boys of the TI group significantly increased their VO_2peak_, while girls of the TI group showed no significant changes after intervention (Figure 3). It has long been established that aerobic MVPA can increase children’s cardiorespiratory fitness [8]. Aerobic fitness requires on-going training of appropriate specificity to be sustained and improved. It also requires specific dosage, frequency, duration, and intensity of the exercise intervention [13,45]. This highlights the importance of an intervention of sufficient quality and quantity to improve children’s cardiorespiratory fitness [46,47]. However, the lack of increase of TI girls’ VO_2peak_ suggested that gender may be a factor influencing the effects of the EO intervention on cardiorespiratory fitness. Boys increased this capacity independently from PE intervention type. Girls seem to profit selectively only from EO intervention, significantly improving their aerobic capacity. Boys are usually more active than girls [6]. Thus, the enhancement of aerobic capacity in boys regardless of PE intervention could be due to their higher practice of aerobic activities in out-of-school settings [48]. The EO intervention seems to compensate the lack of stimulation in aerobic fitness in girls.

Finally, both groups showed an increase of coordinative abilities after the intervention period. These results suggested that both types of intervention were effective to improve motor coordination components such as rhythm, laterality, agility, balance, and strength [49]. However, the efficacy of a wide range of qualitative coordination demands for children’s coordination development was demonstrated by the greater increase in balance beam, jumping laterally and shifting platforms tests of the EO group [15]. The major increase of the balance beam test in the EO group could be attributable to the use of an unstable and irregular surface such as sand [50]. The practice of physical activity on this type of surface could have benefited the sensory system, by providing tactile, visual, and proprioceptive inputs, thus improving children’s balance and motor coordination performance [51]. Similarly, the greatest increase in the shifting platforms test of girls of the EO could be due to the positive effects that the sand surface had on the improvement of the dynamic balance component of this test. Previous studies showed that training on the unstable surface of sand could positively influence balance and jumping abilities development [50,52]. To perform successive jumps into the sand, subjects activate an additional force compared to the force activated to perform jumps on firm ground as gymnasium floor, and over time this seems likely to increase strength [50]. Therefore, the greater increase in the jumping laterally test in the EO group of our study could reflect these increases in muscle strength and power.

Finally, girls performed better than boys in the balance beam test. These results are in line with previous studies that have shown better performance on balance tests in girl children and adolescent girls than boys [5,53,54]. This could be caused by the considerable disparities in pre-pubertal posture between boys and girls, since they have different body heights and weights, which result in different lumbar angles and centers of gravity [55].

### Strengths and Limitations

This study could provide useful suggestions for PE activities in natural environments. It could also significantly contribute to the understanding the feasibility and the beneficial effect of PE in outdoor natural environments, that could be applied in different situations that need to pay particular attention to the dimensions of PE spaces in order to prevent or limit contagions. Moreover, it seemed particularly important in the Italian school context since outdoor PE interventions could remedy the problem of insufficient and inadequate spaces for PE. About 4% of the primary and lower secondary schools do not have a school gym, and this situation limits students’ use of an important space for physical activity [56].

This study had some limitations. Since it included a sample of primary schoolchildren, the findings could not be generalized to samples of different ages. Another limitation resulted from the lack of assessment of further components of physical fitness (e.g., muscular strength and endurance, flexibility, and speed) beyond the aerobic fitness. In the present study, the equation of Matsuzaka et al. [30] was used to predict VO_2peak_ from the PACER scores. Recently, a new approach was implemented in adults to monitor their fitness status by detecting aerobic threshold from HR variability [57,58]. Further research could be conducted to investigate this method as a complementary criterion for testing VO_2peak_ in children and youth. The lack of assessment of time children spent in sedentary activities could be a potential confounder, influencing differently the level of fitness of children belonging to the two intervention groups.

## 5. Conclusions

In conclusion, the results of this study showed that a well-structured PE intervention conducted in the natural environment of the beach could improve children’s physical fitness and gross motor coordination. This study confirmed the positive impact that the practice of physical activity in the natural environment could have on children’s physical fitness, promoting the increased light to moderate physical activity and favoring a wider variety of physical exercises [59]. Moreover, significant effects were found in balance and coordinative abilities. These skills are particularly important for children’s general mastering of their own body in relation to the physical environment [60].

Planning outdoor PE interventions could be an alternative and safe way to encourage and implement physical activity at school, especially during the current period of COVID-19 pandemic, that often required the suspension or reduction of PE practical lessons in many schools. Therefore, this study could represent a new perspective for PE, strongly affected by the pandemic restrictions, promoting the outdoor PE activities as an essential part of PE curriculum.

Although not completely investigated, this study indicates the natural environment of the beach as a potential factor that could positively influence children’s motor development. Further studies should be conducted to verify whether specific motor skills could be better learned in a particular natural environment than in others.

## Figures and Tables

**Table 1:** Number of children belonging to experimental outdoor intervention (EO) and traditional indoor intervention (TI).

	EO			TI		
	Girls	Boys	Total	Girls	Boys	Total
Grade 2 (7–8 years of age)	12	13	25	6	4	10
Grade 3 (8–9 years of age)	8	7	15	10	9	19
Grade 5 (10–11 years of age)	12	12	24	6	7	13

**Table 2:** Pre- and post-intervention values (mean values ± SD) of experimental outdoor intervention (EO) and traditional indoor intervention (TI).

	EO		TI	
Variable	Pre	Post	Pre	Post
Weight (kg)	32.2 ± 10.9	34.2 ± 11.8	34.1 ± 10.0	35.6 ± 10.5
Height (cm)	131.3 ± 18.9	135.8 ± 10.0	134.3 ± 10.4	136.8 ± 10.6
BMI (kg/m^2^)	17.7 ± 4.1	18.2 ± 4.3 *	18.8 ± 3.8	18.6 ± 3.5
%FM	24.3 ± 6.5	24.2 ± 6.5	25.3 ± 6.3	24.4 ± 7.1
Abdominal circumference (cm)	61.4 ± 10.3	62.5 ± 11.0	64.5 ± 10.6	66 ± 10.6
Systolic blood pressure (mmHg)	99.6 ± 14.2	101.7 ± 10.9	105.8 ± 13.7	104.8 ± 10.3
Diastolic blood pressure (mmHg)	60.7 ± 8.3	60.5 ± 6.3	61.7 ± 7.8	62.6 ± 6.9
Resting heart rate (bpm)	77.6 ± 12.3	84.1 ± 10.6 *	83.5 ± 9.9	79.0 ± 11.2 *
PAQ-C-It (score)	2.4 ± 0.6	2.4 ± 0.8	2.0 ± 0.6	2.1 ± 0.8
VO_2peak_ (mL·kg^−1^·min^−1^)	42.8 ± 4.1	43.5 ± 4.7	41.6 ± 3.8	42.4 ± 3.8
Laps (num)	9.9 ± 5.7	16.1 ± 9.2 *	8.9 ± 2.8	12.0 ± 5.5 *
Shifting platforms test (score)	31.2 ± 9.3	39.6 ± 9.3 *	29.6 ± 9.2	36.1 ± 10.7 *
Balance beam test (score)	34.3 ± 16.9	50.1 ± 16.0 *	31.5 ± 15.8	41.7 ± 15.7 *
Jumping laterally test (score)	23.3 ± 16.5	45.7 ± 21.0 *	21.5 ± 12.9	35.0 ± 18.7 *
Hopping on one leg test (score)	37.0 ± 19.1	49.2 ± 18.0 *	35.7 ± 16.8	47.9 ± 16.1 *

* *p* ≤ 0.01 post vs. pre.

## Data Availability

Individual anonymised participant data will not be shared. Pooled study data, protocol, or statistical analysis plan can be shared upon request at mariachiara.gallotta@uniroma1.it.
